# High resolution cine displacement encoding with stimulated echoes (DENSE) at 3T with navigator feedback for quantification of cardiac mechanics

**DOI:** 10.1186/1532-429X-16-S1-P48

**Published:** 2014-01-16

**Authors:** Gregory J Wehner, Jonathan D Suever, Christopher M Haggerty, Linyuan Jing, David Powell, Xiaodong Zhong, Frederick H Epstein, Brandon K Fornwalt

**Affiliations:** 1 Biomedical Engineering, University of Kentucky, Lexington, Kentucky, USA; 2 Pediatrics, University of Kentucky, Lexington, Kentucky, USA; 3 MR R&D Collaborations, Siemens Healthcare, Atlanta, Georgia, USA; 4 Biomedical Engineering, University of Virginia, Charlottesville, Virginia, USA

## Background

Measures of cardiac mechanics such as myocardial wall strain are better predictors of outcomes in patients with heart disease compared to traditional clinical measures and ejection fraction. Cine displacement encoding with stimulated echoes (DENSE) is an ideal method for quantifying cardiac motion which encodes tissue displacement in the phase of the MR signal and provides pixel-level resolution for quantifying cardiac mechanics. To date, DENSE has been implemented with resolution limited to 2-3 pixels across the myocardium. While this resolution is higher than most other techniques for quantifying cardiac mechanics, it may limit the ability of DENSE to quantify finer details such as transmural strains (subendocardial, midmyocardial and subepicardial) and right ventricular mechanics. We hypothesized that it is possible to efficiently increase the resolution of DENSE by a factor of 4 utilizing a navigator feedback system.

## Methods

10 subjects (age 27 ± 3) with normal ECG and no history of cardiovascular disease were consented. A 3.0T Siemens Tim Trio with a 6-element chest and 24-element spine coil was configured with a navigator feedback system. The feedback system projected the navigator image of the diaphragm to the subject in real time to optimize breathold position. Standard resolution 2D cine DENSE was acquired with: 6 spiral interleaves, FOV = 340 mm, matrix = 96 × 96, thickness = 8 mm, TE/TR = 1.08/17, flip angle = 20, averages = 1, navigator acceptance window = ± 3 mm. High resolution 2D cine DENSE images were acquired by quadrupling the number of spirals to 24, increasing the matrix to 256 × 256, and increasing the averages to 3. Three short- and two long-axis images were acquired with each technique. Left ventricular strains and torsion were compared between the techniques using Bland-Altman.

## Results

**Figure 1:**
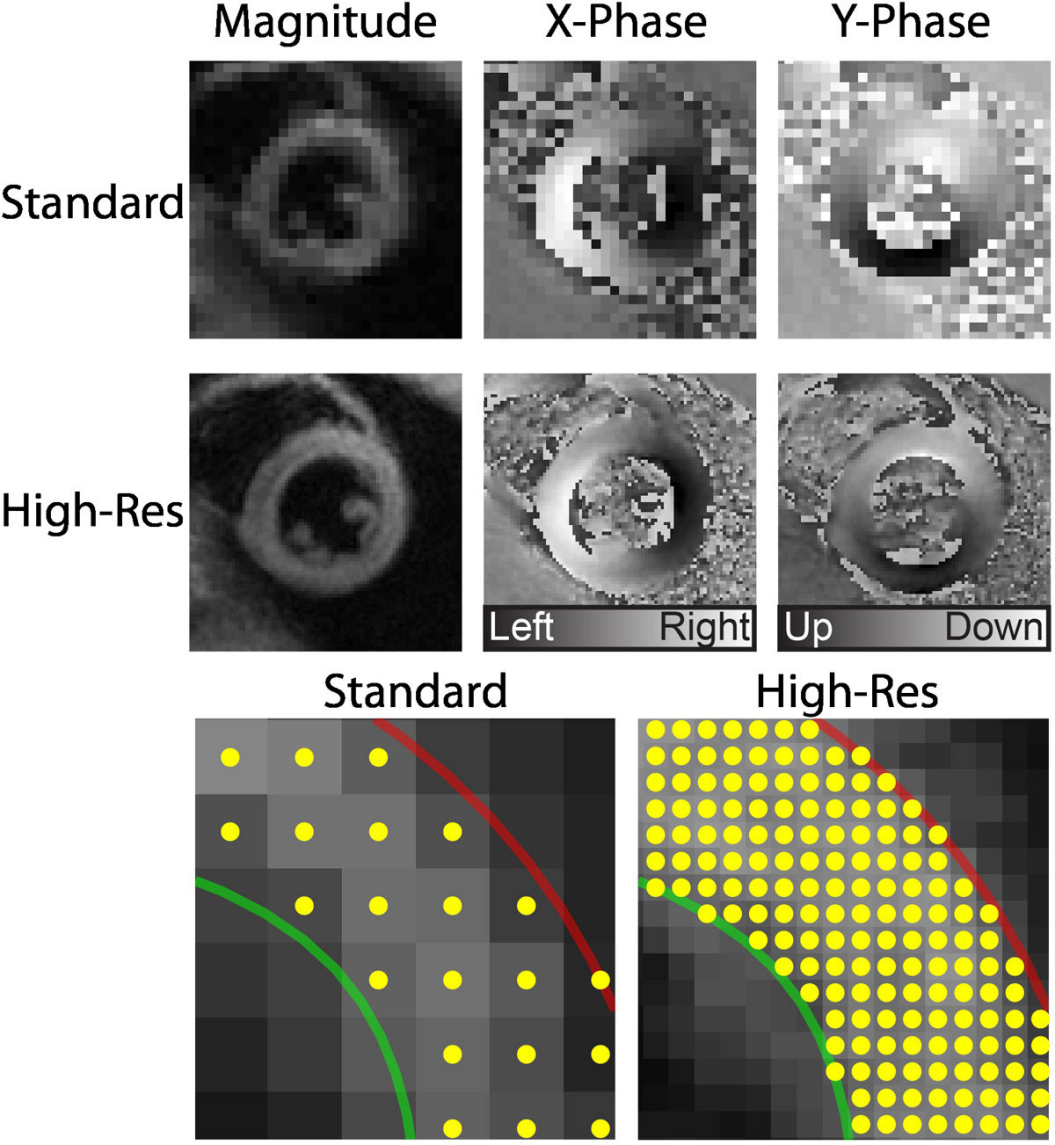
**Comparison of standard and high resolution DENSE MRI**.

The high resolution images took 11 times longer to acquire but the navigator feedback system provided good efficiency (69 ± 9%) for a total acquisition time of roughly 5 minutes per slice. The high resolution images had excellent quality with a noticeable improvement over standard resolution (Figure 1). There was a systematic but negligible difference between standard and high resolution data for circumferential and longitudinal strains (Table 1). Radial strains showed the largest differences consistent with a systematic under-estimation of radial strain from standard resolution DENSE. Torsion was not significantly different between the two methods.

**Table 1:** Strain and torsion calculated from standard and high resolution DENSE MRI

	Standard Resolution	High Resolution	Bias (Mean Difference)	95% Limits of Agreement
**Circumferential Strain (%)**				

Transmural average	-20 ± 4	-19 ± 4	-1	± 3

Subepicardial	-15 ± 3	-15 ± 4	-1	± 2

Midwall	-20 ± 4	-19 ± 4	-1	± 3

Subendocardial	-25 ± 4	-23 ± 4	-2	± 4

**Radial Strain (%)**				

Transmural average	35 ± 12	45 ± 13	-10	± 21

Subepicardial	38 ± 13	44 ± 12	-6	± 20

Midwall	41 ± 13	52 ± 18	-11	± 30

Subendocardial	23 ± 15	36 ± 18	-13	± 27

**Longitudinal Strain (%)**				

Transmural average	-15 ± 2	-13 ± 3	-2	± 5

Subepicardial	-15 ± 2	-14 ± 3	-1	± 5

Midwall	-15 ± 2	-13 ± 3	-2	± 6

Subendocardial	-16 ± 2	-13 ± 3	-2	± 6

**Torsion (degrees/cm)**				

Transmural average	3.5 ± 1	3.5 ± 1	0.1	± 1

Subepicardial	2.7 ± 1	2.7 ± 1	-0.1	± 1

Midwall	3.5 ± 1	3.5 ± 1	0.0	± 1

Subendocardial	4.5 ± 1	4.1 ± 1	0.3	± 1

Strain and torsion values are reported as mean ± standard deviation. Bland-Altman 95% limits of agreement are provided for the bias.

## Conclusions

High resolution cine DENSE MRI with navigator feedback is feasible at 3T and produces high quality images with 4 times the resolution of standard DENSE. Left ventricular circumferential strains, longitudinal strains, and torsion showed negligible differences between high and low resolution DENSE. Radial strains were significantly different, potentially due to better accuracy with high resolution DENSE due to the increased number of pixels within the thickness of the myocardial wall.

## Funding

NIH Interdisciplinary Cardiovascular Training Grant (T32 HL072743) NIH Early Independence Award to BKF (DP5 OD012132) University of Kentucky Cardiovascular Research Center, grant UL1RR033173 from the National Center for Research Resources (NCRR), funded by the Office of the Director, National Institutes of Health (NIH) and supported by the NIH Roadmap for Medical Research Contributions made by local businesses and individuals through a partnership between Kentucky Children's Hospital and Children's Miracle network The content is solely the responsibility of the authors and does not necessarily represent the official views of the funding sources.

